# Exploring adversities among parents convicted of killing their children

**DOI:** 10.1371/journal.pone.0235812

**Published:** 2020-07-22

**Authors:** Bianca Dekel, Michelle Andipatin, Naeemah Abrahams

**Affiliations:** 1 Gender and Health Research Unit, The South African Medical Research Council, Cape Town, Western Cape, South Africa; 2 Psychology Department, University of the Western Cape, Cape Town, Western Cape, South Africa; Sapienza, University of Rome, ITALY

## Abstract

Experiencing adversities has been associated with the use of violence but this has not been explored with filicide offenders in South Africa. Individual, semi-structured interviews were conducted with 22 parents/stepparents/caregivers convicted of child homicide in South Africa, resulting in 49 in-depth interviews. Data were analyzed by means of grounded theory. Using an ecological framework, this study alludes to the widespread and cumulative nature of violence and trauma experiences within multiple domains of the participants’ lives. The study highlighted the absence of support in the aftermath of experiencing trauma, possibly resulting in these parents lacking resources to mitigate the sequelae of adverse experiences. This study calls for trauma related, mental health components to be integrated into violence interventions and for these to address the impact of trauma at the individual, family, and societal levels, to prevent the transition from victim to offender.

## Introduction

Child homicide by a parent is an unfathomable crime and has received poor research attention in developing settings. To assist in understanding this phenomenon, Resnick, who was trained as a psychiatrist, developed the first filicide classification system in 1969, structured according to the apparent motive for the killing. This classification system was developed after an extensive global literature review and analysis of 155 child murder studies published from 1751 to 1967. The following six categories were established [1]. Altruistic filicide refers to instances where the motive is to relieve the child of real or imagined suffering. It is also usually associated with suicide, where the parent believes they cannot abandon their child(ren) when they commit suicide. Acutely psychotic filicide involves parents who kill under the influence of severe mental illness. In unwanted-child filicide, the victim was never or is no longer desired by the parent(s), and in accidental filicides, the death is an unintentional death (homicidal intent is lacking) due to fatal child abuse. Spousal revenge filicide describes children who are killed in the parent’s attempt to retaliate against or punish his/her current or ex intimate partner [1].

Neonaticide was Resnick’s [2] final category. He was the first to distinguish the age differences between children killed, and used the term neonaticide, to define “the killing of a neonate on the day of its birth” [2, p. 1414]. He reasoned that the motives, circumstances of the killing, and psychosocial background of neonaticide offenders are different from those found amongst parents who kill older children. He found neonaticides tend to be committed either because the infant is unwanted, there is extramarital paternity, the infant was conceived out of rape, and/or the child is seen as an obstacle to parental ambition. He also noticed that these pregnancies tend to be kept secret from family and friends [2].

Further, the cases upon which Resnick’s [1, 2] system was founded, originated from sources and cultures which may not apply to parental child homicide in many settings, such as South Africa. The common ways in which to kill a newborn reported by Resnick [2, p. 1415] included using “acid, lye” or “throwing” the newborn “to pigs”. Many of these methods represent cultural or temporal distinctions that are not relevant to a contemporary South African context.

Nevertheless, within South Africa, parental child killing is not well understood. International research suggests that filicidal parents tend to have trauma histories, which may contribute to their use of violence toward their children [3]. Experiencing adversities/trauma may influence violence perpetration [4, 5] as it may increase the possibility of developing mental/behavioral dysfunction [6]. Biological explanations point to changes in neurobiology post-trauma, with survivors struggling to think logically, reactions reflecting poor impulse regulation, and inappropriate expression of anger. This may lead to engagement in violent behavior [7]. Repeated violence exposure may also lead to desensitization, often contributing to decreased empathy, which may add to feelings of aggression [8].

Research has also found that it is not uncommon for trauma to lead to adjustment and behavioral problems, which may evolve into antisocial behavior. Research with male and female juvenile offenders, found trauma experiences and victimization in their family of origin, was salient to elucidating their pathway to crime [9]. Indeed, the link between multiple trauma and violence perpetration has been observed in several studies with incarcerated individuals [4, 10, 11].

Expectedly, research has found the presence of mental illness amongst parents who kill their children [e.g. 12, 13, 14, 15]. Evidence indicates that parents with adverse mental health (e.g. experiencing depression or PTSD related symptoms) tend to experience difficulties in parenting. To illustrate, a United Kingdom based cohort study, found such parents were less responsive and less positive toward their children. The study also found maternal depression was associated with the use of harsh disciplinary practices [16]. Psychiatrists [17] proposed that trauma symptoms, such as depression and anxiety, may result in an inability to hear children’s distress. It may also result in a parent’s need to withdraw to protect themselves from feelings of vulnerability and may therefore be intolerant to their children’s resultant anxiety and aggression.

In line with this, researchers studied the detrimental impact of cumulative trauma experiences on the mothering role [18, 19]. They found trauma exposure was linked with decreased parenting satisfaction, reports of child neglect and abuse, and a history of protective service reports. This emerging literature on trauma and parenting confirms longstanding notions that a history of abuse can negatively affect a parent’s caretaking abilities in ways that can have far-reaching consequences.

South African research has shown that violence is common in many settings i.e. within the workplace, communities, home, school, and politics [20]. South Africa has a long history of violence and underlying contributing factors are complex and there is no single cause but rather, a plethora of intersecting factors, which combine to give rise to these high levels. There is no doubt that the violent past of legalized, racial inequalities and subordination of the majority of the South African population is the foundation for the current levels of violence [21, 22]. Since the demise of apartheid, violence has largely become normative as it is viewed by many as an appropriate means to settle disputes [23], giving rise to South African’s levels of violence, homicide, sexual violence, and violence against children [24]. It is almost anticipated then that many people are exposed to multiple instances of violence within South Africa, with high rates of poly-vicitmization [25]. For example, the South African Stress and Health study, a nationally representative, epidemiological survey of 4351 adults showed that South Africans tend to experience multiple traumas and that these high trauma rates appear to be taking a serious toll on South Africans’ psychological health [25].

Using the ecological approach [26], the South African Stress and Health Study showed how experiences of trauma occurred in multiple domains (e.g. within the family/relationship domain, such as the death of a loved one and experiencing abuse by a partner, and within the community, such as criminal victimization) [25]. This approach, therefore, provides a framework for understanding the possibility of a parent becoming abusive towards a child as a function of individual, relationship, community, and societal factors, intersecting at multiple domains of the social ecology. This framework is inclusive of individual’s life histories, including traumatic scars, which a father/mother possibly brings to their relationships with their children, as well as the context, and situational factors that influence their everyday lives. The ecology also includes social norms (shaped by structural factors and ideologies), which are reinforced across different social settings. For example, the shaping of parental behavior rooted in the acceptability of violence to discipline children [27]. However, Galtung [28] reminds us that the role of structural violence is not meant to weaken our ability to hold individuals/parents accountable for their actions, but rather to enhance our capacity to more clearly understand the manner in which structural violence plays out beyond the personal to also include structural and cultural sources of violence. It is therefore no surprise that violence against children is part of the violence experiences in South Africa with child homicide the most extreme form of violence against children reported at excessive rates compared to other countries [29]. A study reflecting on 2009 data reported a child homicide rate of 5.5/100,000 population [29] which is more than double the global rate (2.4/100,000) (UNODC, 2013). This study also noted that a parent was the most common perpetrator [29]. It is important to acknowledge here that although both parents commit such acts, we are to be mindful that mothers should not always be constructed as passive and powerless victims, suggesting that the power dynamics are far more complex, playing out differently in various contexts [30]. Consequently, we acknowledge that there is a tendency to remain within a relatively ‘safe’ and familiar realm by explaining women and men’s violence through emphasizing female victimhood/female passivity and male oppression/male power, instead of exploring the potential for agency [31]. Thus, we acknowledge that parents’ use of violence may involve a degree of choice from within a range of possible choices and therefore, one could ask whether murdering a child could be interpreted as a form of agency, whether consciously or unconsciously, in the circumstances of the lives of these parents. We are to be mindful that context enables and produces, but does not determine crime, and its consideration need not negate agency and responsibility [31].

Very little is known about the life histories of South African filicidal parents. This paper draws on data from a qualitative study with parents convicted of killing their children in South Africa and uses an ecological approach to understand how previous trauma experiences may have contributed to the use of violence against their children.

## Methods

Ethical approval was obtained from the Humanities and Social Sciences Research and Ethics Committee at the University of the Western Cape and the South African Medical Research Council (Protocol ID: EC008-5/2018).

### Participants and instruments

A qualitative study, using individual, semi-structured interviews, was undertaken with 22 parents (all names used are pseudonyms) (Table 1) incarcerated for the murder of a child. The parents were recruited from five correctional centers in the Western Cape Province of South Africa. The inclusion were: Mentally sound parents (biological fathers, biological mothers, stepparents, and/or caregivers), who were over the age of 18 years at the time of the interviews, and who were previously convicted of the murder of their child(ren) aged 18 years and below. The life story approach to data collection was adopted with participants asked to narrate their life stories. Repeat interviews were done for most participants to allow for building of rapport and opportunities to clarification. An example of a question asked during the first interview included: “Tell me about your childhood life” and second interview is: “Tell me about the events leading to your child’s death”. Interviews ranged between one to two hours and a scope of enquiry guided the interviews.

**Table 1:** Sample characteristics using pseudonyms.

Name	Age	Race	Victim age	Relationship to victim
Lauren	28	Black	Newborn	Mother
Winnie	20	Black	1 wk old	Mother
Jennifer	29	White	3 mnths old	Mother
Abigail	34	White	6 mnths old	Stepmother
Christelle	36	Colored	6 mnths old	Mother
Patricia	32	Black	1 yrs old	Mother
Deidre	22	Colored	1 yr and 2 mnths	Mother
Michelle	23	Colored	2 yrs old	Caregiver
Zubeidah	34	Colored	2 yrs old	Mother
Nicole	34	Colored	2 yrs old	Mother
Ryan	32	White	2 yrs old	Caregiver
Zolu	33	Black	2 yrs old	Father
Sipho	30	Black	2 yrs old	Father
Howard	33	Colored	2 yrs old (twins)	Stepfather
Thandi	31	Black	3 yrs old	Mother
Latifa	36	Colored	5 yrs old	Stepmother
James	36	Colored	5 yrs old	Stepfather
Cayleigh	32	Colored	7 yrs old	Stepmother
Jamaal	38	Colored	8 yrs old	Father
Nelly	41	Black	9 yrs old	Mother
Adam	45	Colored	12 yrs old	Father
Michael	53	White	21 mnths old, 5 yrs old, 16 yrs old	Father

### Procedure

Access to correctional centers/participants was granted by the Department of Correctional Services and data collection was conducted between April–December 2015. Fourteen participants were recruited using purposive sampling. Correctional center psychologists identified incarcerated parents and asked each whether he/she would be willing to participate. An additional eight participants were identified through snowball sampling with the first set of recruited participants providing names of offenders they knew who met study inclusion criteria. Informed consent procedures were done before interviews with detailed explanations of the processes and risks involved. The first author (who has a psychology degree and has qualitative and gender-based violence research experience) was the sole interviewer and was accompanied by one of two translators (Afrikaans and isiXhosa) if the participants requested for the interview to be done in a mother tongue language. Two participants required the assistance from the isiXhosa translator and five participants conversed in a combination of English and Afrikaans, where the Afrikaans translator aided at times, and the remaining 15 participants spoke English. Thus, interviews were conducted in the preferred language of the participant and interviews were recorded, translated, and transcribed into English. Since the study was conducted in a correctional center setting, an incentive was not provided. We arranged for psychological support post-interviews with correctional center psychologists, and five participants were referred for counseling. Counseling was also available for the first author and translators.

### Data analysis

The qualitative software program Atlas T.I version 8.0 was used to assist in the organizing of the analysis of the data [32]. During open coding, audio-recordings and transcripts were reviewed and assigned codes. Emphasis was placed on allowing concepts to emerge without forcing them into predefined categories. Categories were divided into sub-categories as 108 codes were created and trimmed down to 54 codes. The second stage involved identifying a single category as the central phenomenon, which was positioned as a central feature, and relationships between categories and sub-categories emphasized. In the last step, a storyline was constructed and discussed with co-authors, and member checking was performed with participants.

## Results

The study included 14 female and eight male participants, responsible for the death of 25 children. Twenty participants killed one child each and two participants (one father and one stepfather) killed more than one child (three and two children respectively). The sample encompassed mothers (10), fathers (4), stepfathers (3), stepmothers (3), and caregivers (2). Participants were racially categorized as Colored (11), African (7), and White (4) (using the apartheid racial classification system) and were aged between 20 and 53 years old. The children killed ranged in age from newborn to 16 years old. The predominant manner of death was fatal child abuse. Additional information is provided in Table 1. The participants received sentences ranging from eight months to life. Of the 22 participants, six had prior convictions, ranging from robbery to rape. The majority of participants grew up in communities marked by hardships, including widespread violence, crime, and gangs, poor policing, unemployment, drug abuse, and domestic violence.

### Individual level

#### Adverse parenting experiences

The majority of participants [20] experienced traumatic parenting practices of abuse, neglect, and abandonment. These narratives are explained in detail in a separate paper [33]. The data showed relationally based, individual level factors were salient to elucidating pathways to crime. For example, parental abandonment and, thus, displacement from one home to another during childhood, was a frequent theme within 13 participants’ narratives. Participants explained these moves were traumatic as it signified a loss experience as they were forced to leave behind caregivers who they had formed close bonds with. To illustrate: “I missed my mother so much when I moved to my grandmother” (Lauren: mother: killed newborn). For many participants, these moves played a role in leading to deteriorated school performance. For example, Jennifer (mother: killed three month old) said: “I struggled to concentrate at school” and Deidre (mother: killed 1 year 2 month old) mentioned: “I did drop out of school”. The male participants sought affirmation from peers through gang memberships (outlined further on) which included the use of drugs and alcohol.

#### Substance use

Alcohol and drug misuse were common amongst 17 participants who linked their substance use with attempts to cope with trauma, which also likely increased their propensity for violence. For example, at the age of nine, Michelle (caregiver: killed 2 year old) was raped and reported: “That’s why I started to use drugs…I tried to cover it up…the drugs made me forget”. Similarly, to deal with her mother abandoning her, Deidre said: “When I think about my mother, then I just go smoke. I was putting drugs on top of it”.

Furthermore, another participant’s narrative depicted possible psychopharmacological effects as Nicole reported increased irritability as a result of withdrawal symptoms saying, she felt: “grumpy”, would tend to “swear at everyone”, and also admitted that if her son cried, she would be “rough with him”.

Un-coincidently then, most participants [14] reported being intoxicated at the time of their crimes. Jamaal (father: killed 8 year old) said: “I was high” and Zubeidah (mother: killed 2 year old) said: “I was using drugs non-stop from the day before”. Most participants reiterated Zubeidah’s sentiments, that: “if I was sober, my child would still be alive”, which may seem they blamed alcohol or drugs for their actions, thus, providing a means for them to negate responsibility.

### Family/relationship level

#### Intimate partner violence

Abuse was endemic both within their parental and romantic relationships. Six men reported perpetrating Intimate Partner Violence (IPV) and 11 women were in abusive relationships. Detailed narratives of perpetration of and victimization through IPV are outlined in a separate publication [34].

Their narratives showed that exposure to IPV started as children, when they witnessed violence between parents, which possibly influenced their own perpetration and victimization as adults. For example, Adam (father: killed 12 year old), in referring to his stepfather, said: “he used to hit my mother in front of us, it was nothing new” and as an adult, Adam explained he: “smacked” his wife if she upset him. Similarly, many women witnessed their fathers abuse their mothers and as adults, were also in abusive relationships. One woman reflected on her cumulative exposure to abuse: “I saw my dad hit my mom. Then my mother left us, and then when I was older, my stepmom did the same to me and then my boyfriend did do the same to me, they both beat me up” (Deidre).

#### Violence affecting loved ones and losing loved ones

It, thus, became evident that most [17] participants were exposed to traumatic, violent experiences within their family of origin, possibly desensitizing them to violence from a young age and inducing some degree of post-traumatic stress. Adam reported his father: “was murdered” and at the age of 10, Deidre witnessed a man: “throw my father with a brick against his head and blood was rushing out. I can never forget that”. Similarly, Ryan (caregiver: killed 2 year old) was exposed to his father and his friends who: “were very violent and fought a lot…and I thought I wanna be like them”. A few years later, Ryan’s parents separated, and his father lost contact with him. Afterward, his maternal uncle played an influential role in his life, but soon thereafter also: “went to prison”. Thus, participants experienced direct losses through imprisonment and death of people close to them, another traumatic experience.

Despite being able to identify how their losses caused pain, and influenced their behaviour, they were seldom afforded opportunities to grieve. It appears that no one in their environment noticed their degenerating behaviors and, thus, did not associate this behavior to their grief. For example, two men, Ryan and James (stepfather: killed 5 year old), both lost their only siblings as young adults (19 years old), which had emotional consequences for them. Ryan described his reaction to his brother’s death: “My world shattered…I didn’t care to live anymore…When he passed I started drinking and I got involved with a bad crowd”. The traumatic experience continued for Ryan when his parents separated afterwards, and his father abandoned him, resulting in him delving deeper into his addiction and delinquent behaviors.

Similarly, James also experienced a previous loss during childhood (aged four) when his mother passed away. It is, however, the death of a second close family member: his brother, which affected him most. He explained: “My life changed like really going downhill when my brother passed. We were very close…After that, everything fell apart…My heart died but my brain was working…I started getting involved with the wrong crowd after that”. James’s father was unable to provide emotional support due to his own alcohol addiction. In each of the narratives of loss, there is a description of deterioration in behavior. There is also an absence of any reference to interventions from caring adults to recognize this distress. Instead, it appeared parents were incapable of support, as James explained: “My father would run away when I tried to talk to him about how I felt”.

The losses they experienced, and the cumulative adversities, possibly resulted in both men displaying signs of mental instability. It is possible to develop appetitive aggression with cumulative trauma experiences, whereby the victim learns that acting aggressively can entail the advantage of no longer being a victim [35]. Appetitive aggression may, thus, reduce chances of developing Post Traumatic Stress Disorder (PTSD) [36]. James’s exchange (in speaking about his five year old stepson whom he beat to death) reflected possible callous-unemotional traits, which may be connected with a lack of remorse and lower levels of PTSD post-perpetration. James explained: “Nothing inside of me feels bad or regret for what I did to that kid…My heart is hard…I don’t get nightmares…I don’t care…You don’t have to do me wrong. If I want to hurt you I do it with the aggression… Kill or be killed”.

#### Sexual violence

Participants were not only exposed to trauma and violence within their family of origin, but traumatic experiences of sexual violence were also reported. However, this was not reported by male participants, but were reported by nine women. Jennifer was raped at six years old and explained that it led to her struggling to cope academically and as an adult she engaged in sex work and: “was a drug and alcohol addict”. Poverty compounded the women’s’ traumas of sexual abuse. For example, at age 17, Winnie (mother: killed 1 week old baby) was raped by six men, which resulted in a pregnancy. She killed her one week old infant because: “she did not have clothes…so I decided instead of her being needy, it is better that I kill her”. Winnie received no support (from family or support services) after the rape. Together, this possibly sheds light on how traumatic events (e.g. rape), coupled with economic marginalization, may engender internal reactions (e.g. PTSD), which may culminate into externalizing behaviors (e.g. violence perpetration).

#### Traumatic work-related violent events

Just as sexual violence experiences were not reported by men, traumatic work-related experiences of violence were not reported by female participants but were noted within three of the men’s narratives. These experiences were within the apartheid era, a traumatic time in South Africa, characterized by structural, political and racial violence [20]. Here we focus on Adam as an example. He enlisted into the apartheid-era, highly militarized, South African army, at the age 20, and witnessed and participated in many atrocities in the form of the war against the resistant movement.

His narrative is illustrative of a man who cannot rid himself of the ghosts that haunt him: “I kill a lot of people while in the army…The last one that I shoot was a lady…She was begging but I didn’t have mercy. I kill her…But now, I still have nightmares. This is the price I pay, because she begged not to kill her. At night before I go to sleep, I picture her face and her begging for me not to shoot”. Adam’s narrative alludes to unresolved trauma, which may have contributed to his drinking and violent behavior. He was convicted of murdering his daughter. The manner in which he describes the killing, echoes army training: “She was sitting in front of me…I grabbed her…when I strangled her, I didn't think about stopping… I put more force in my arms”. Likewise, within the army, Adam was taught to: “fight and not stop, just continue, doesn’t matter if it was a woman [he abused his wife] or a young person [he killed his daughter]. When I fight, I’m taught to see nothing in front of me but the person I’m fighting and to hurt you”.

### Community level

#### Gang involvement

Four male participants reported gang involvement from young ages (e.g. 15 years old). Linking up with gangs resulted in them dropping out of school and an involvement with the drug trade, the latter a possible route out of poverty, as employment was difficult. Peer group influence combined with family dysfunction resulted in Jamaal leaving home at the age of 13: “to live on the streets” after which he: “started smoking glue and breaking into cars….and became a gangster…started robbing people”. It is possible that these adversities, within their family of origin, such as being abused (e.g. “my stepmother did stab me” (Jamaal)), possibly led these men to seek a life elsewhere.

Living on the streets is one possible pathway to violence for men. The street code requires men to adopt presentations of self that demonstrate willingness to use violence. James explained: “I was abused at home so I run away to live on the streets…That is where I get involved in crime…The street life was better than being at home…I’m not evil…I develop this aggression on the streets: for not caring for people, if I want to hurt them, I hurt them…I had to learn to protect myself because the streets is tough and nobody looks out for you besides you”.

Seven men also endured traumatic experiences of an absent male role model, which possibly led them to seek relationships outside of the home. For example, Jamaal explained: “I don’t get the love I needed from my father, so I’m rather going to participate in gangsterism, where I get love and respect”. Being a gang member possibly reduced their feelings of loneliness, but they had to prove their worthiness to be accepted and respected by using violence. As gang members they witnessed (e.g.: “beat him to death”), perpetrated (e.g.: “I took out my knife and I stabbed the one guy to death”); and experienced extreme violence (e.g.: “I was shot”).

Some individuals may develop PTSD after committing violent acts. However, the notion of perpetrator PTSD (alluded to in Adam’s narrative outlined earlier) is not meant to exonerate perpetrators. The potential role of trauma in gang membership is demonstrated by Howard (stepfather: killed 2 year old twins): “While I was a gangster I committed murder…I take it day by day but it's not easy because when I close my eyes it still replays in my head”. Some men displayed symptoms of emotional numbing, which may represent a maladaptive strategy for coping with trauma. James explained: “I’m used to violence…it’s nothing for me to see someone die…because I have seen it every day. I’m a part of it now”.

Belonging to a gang requires members to adhere to standards of behavior. If a member: “steps out of line” (James), he is punished: “beaten up or killed” (Jamaal). James may have viewed his stepson as stepping out of line and his violent response was possibly in keeping with the standards of his gang which he joined at the age of 16. James argued his stepson did not: “listen” to him: “if I ask him to do things for me” which was: “embarrassing”; the latter may imply shame related to the inability to control a child, and possibly a sign of weakness, which required a display of aggression, in order to adhere to notions of hegemonic masculinity that he espoused. Consequently, James does not view himself as: “an evil person” for killing his stepson and fails to view his response as an act of violence but as a response to being provoked.

The reason why some men use violence as a means to secure control may lie in their structural positions, and their loss of status—(and power) in not being able to live up to the masculine ideal of being a provider. According to James, his stepson reminded him of his mother-in-law (stepson’s grandmother), explaining that: “I started to get angry, because if I see his face, I see his grandmother”. This possibly reminded him that he was unemployed; living with his mother-in-law and relying on her for support. He reported she: “rubbed it in my face that I don’t have money” and that this was: “extremely frustrating”.

#### Violent neighborhoods

Nineteen participants were raised in some of Cape Town’s most violent communities, where trauma exposure is a daily occurrence [37]. Community violence had a direct impact on one participant, Lauren, who was convicted of murdering her newborn by slitting his throat. She said: “I saw blood on the streets, because they used to fight with knives. It was there I learned the knife can be used as a weapon and not just for cooking”. Zolu’s (father: killed 2 year old) childhood also included exposure to violence when he witnessed community members enforcing punishment: “They beat a guy and took a big stone and put it on top of his head and then he died. I watched them drop that big stone on his head”.

#### Health/social services

As this article has shown, participants experienced a surplus of traumatic events prior to killing their children; yet, none received psycho-social support. Many participants reiterated James’s sentiments that: “you are the first person I have spoken to about this”. Their excerpts, as shown in this article, were often possibly indicative of unresolved trauma (e.g. “I still have nightmares”—Adam) and participants were often emotional when reflecting on trauma perpetrated by others, especially by parents. For example, Howard reported: “I’m still so hurt…my father hurt me the most emotionally” and likewise, Patricia said: “My heart was very sore, deep down I am emotionally wrecked by my mother”.

### Societal level

#### Ideologies of culture: Preserving male honor and violent, constructions of masculinity

Norms related to being a man and male honor were instilled in seven of the men from young ages, which possibly shaped their violent behavior. Although James’s father was present and Zolu’s father was absent, both were taught to fight when encountering conflict. James explained: “My father told me, when I come inside, and the kids hurt me…he would say: “go out and fight them. Don’t come back here and cry and tell me about kids hurting you””. Zolu also elaborated: “When you come home crying, the men in our family beat you up and they say: “go fight, why you come home crying?””. The possible impact of these harsh parenting practices was explained by Zolu when he described his desensitization to violence as an adolescent: “I saw a car hit someone dead…I just look and pass…I don’t have feeling. It’s the way I grow up…I was too young to see and go through those things. So I grow up hard…If you see something you never saw, you will be scared but if you used to see it, you don’t worry about it”.

#### Political experiences of violence

Most participants [21] grew up during a traumatic era in South Africa, the 1980’s, characterized by partly legalized, racial violence. None of the women reported traumatic apartheid experiences; however, for some men, apartheid-related violence, was the first type of violent trauma they were exposed to. For example, a seven year old Jamaal endured a traumatic, violent, apartheid experience. He explained: “One of the police guys…assaulted my uncle. That is how I became introduced to violence…I saw blood running so he told me “run” and the cops shot me with a rubber bullet”. He explained this traumatic memory: “never left me” and it would often: “jump into my mind”, once again alluding to the notion of unresolved trauma.

## Discussion

This paper sheds light on how cumulative adversities across the life span overlapped across multiple contexts, which may have influenced violent behavior against children. To the best of our knowledge, this is the first South African qualitative study exploring multiple adversities in the lives of parents convicted of child homicide.

Our findings overlap and confirm results from studies addressing adversities/trauma in the pathway to violence, which found the presence of unstable childhoods, childhood abuse or abandonment, and witnessing parental and community violence [38], PTSD [39], and parental and participant substance use [4]. This paper also confirms prior work on childhood adversities, where the authors [40] found that the onset of drug/alcohol abuse often approximately corresponded to the time of traumatization in their lives, therefore, reporting that these substances decreased their trauma symptoms.

This paper also alludes to the cycle of violence theory, which proposes that victimized children grow up to victimize others [41, 42]. We do acknowledge that human behavior is too complex to isolate certain factors as causative. Yet, in many studies, early traumatic experiences emerge as a strong factor important in determining later development [43]. Yet, history is not destiny. Whether parenthood becomes a repetition of the past in the present [44] is increasingly determined by additional external factors, which contribute to the development of violent behavior amongst parents. Thus, this notion is not without criticism as some researchers [45] argue that most people who experience abuse in childhood do not proceed to abuse their children. In contrast, advocates of this theory suggest that when abused children become parents themselves, they tend to model their parenting behavior on what they observed and learned as children. Thus, early adversity is believed to be a risk factor, at least for some, for maltreatment of one’s own offspring [46].

The literature on transgenerational transmission of trauma and violence further argues that the experience of trauma and violence, particularly if it occurs in early developmental stages, could repeat itself—a process termed re-victimization. For instance, a prospective cohort study conducted over 35 years found individuals who were abused during childhood were at increased risk for experiencing violent re-victimization as adults. It was suggested that abused females may become attached to men who victimize them, whereas abused males are thought to externalize and victimize others. Situational factors may also influence re-victimization through continued residence in impoverished or dangerous areas. However, the mechanisms which place these children on a path toward re-victimization are still not fully understood and warrant further attention [47].

Thus, although a large part of the narratives were about childhood adversities, for many participants, such adversities did not end in young adulthood, as was seen for example, by the abusive experiences reported by the women. Based on this discussion, it is evident that a multitude of factors seemed to influence the pathways to violence. Through the life stories presented in this paper, it becomes evident how the participants’ experiences of childhood abuse and abandonment, combined with unstable childhoods and an absence of supportive adults, their relationships with parents and witnessing parental violence, their exposure to violence, and lack of reparative environments, possibly interacted to inform their use of violence.

Participants had also lost close family members, some to violence. They grew up in violent communities and to cope with problems (e.g. poverty), they turned to substances. Their addictions often meant dropping out of school, leading to unemployment, locking them in the same poverty they grew up in, and for some, their addictions led them to engage in criminal activities to obtain drugs. They searched for love and affirmation they were often unable to receive at home, within gangs, and with those who used violence. Their participation in gangs, drug sales, and violence were ways of coping with familial, social, and economic exclusions. For the men, some of the male influences in their lives encouraged their use of violence (leading some to want to emulate their role models) to resolve conflicts. Indeed, the then political violence did not teach them differently and a general social attitude viewing violence as an acceptable means to solve problems possibly compounded issues. In sum, the stories narrated here show these participants’ experiences of adversities and violence were not restricted to discrete settings and each of these experiences possibly had a cumulative effect.

This study shows that the terms ‘victim’ or ‘perpetrator’ are not dichotomous. Our findings highlight that the children who become violent parents were mostly victims themselves, of violence, and multiple adversities/trauma. While this is not to suggest that these parents are blameless for their deadly actions, it does imply the need for a more compassionate response. For these parents, traumatic events occurred in close proximity and were preceded and followed by additional trauma. If we consider the devastation of a single traumatic event, it becomes unfathomable to picture how one recovers from a series of such events, which take place in rapid succession. For these men and women, returning to a path of non-violence (if they ever had the opportunity to walk such a path) would be almost impossible as they lacked positive resources for mitigating sequelae.

Indeed, this study has shown the consequences of ignoring post-trauma care in the aftermath of violence. The mental health treatment gap is a serious and under-recognized issue within South Africa [48, 49]. This is despite the health burden associated with poor mental health, which invariably impacts the most on those dependent on the public health system. Taking into account Galtung’s work on violence, we are reminded that there is a need to focus on what he terms, ‘indirect harms’ when attempting to understand violence. In other words, ignoring post-trauma care in the aftermath of violence, may lead to avoidable suffering, coping issues and may eventually impact on child abuse and even homicide [28]. Thus, evidence-based interventions tested in poor resource, conflict settings has shown effectiveness in managing PTSD, and a call has been made to test these with community health workers in the South Africa [50]. Similarly strengthening school-based programs to respond to children and youth presenting with trauma related symptoms has also been suggested as part of a comprehensive package to trauma care in the country [39].

The study has limitations. Perpetrator narratives suggest methodological concerns about the ‘truth’ of their testimonies. This study relied on participants’ accounts of their ‘truths’ about their lived experiences. differing perspectives of the crimes. Narrative accounts are not mirrored images of experience and the aim was not to produce accurate recalls but to provide an occasion for reflection on the meaning of these events [51]. In this paper, emphasis is placed on attempting to accept and encourage awareness of contextual factors, as context enables and produces, although it may not determine crime, and its consideration need not negate agency [31].

## Conclusion

This study has shed a light on the exposure to adversities/trauma amongst filicide perpetrators and the possible outcome of not treating the post mental health symptoms. It also illuminated how trauma can possibly lead to problems later on, especially within the parenting role. Trauma has the potential to have a profound impact on parent-child bonds and this study has highlighted the importance of recognizing early adverse experiences on later adult violent behavior. We need to acknowledge the impact these experiences have on an individual's ability to adopt a paternal or maternal role. It is therefore, vital to strive towards reducing exposure and experiences of trauma and/or implementing sufficient interventions post-trauma, in order to promote less violent parent-child relationships and to resolve parents' traumatic experiences.

## Supporting information

S1 File (DOCX)
