# Supplementary material for: Exploring adversities among parents convicted of killing their children
Source: PLoS One. 2020 Jul 22;15(7):e0235812. doi: 10.1371/journal.pone.0235812 (PMC7375607; doi:10.1371/journal.pone.0235812)
Supplement: S1 File — (DOCX) [file pone.0235812.s001.docx]

**Supporting Information**

**Lauren:**

“I missed my mother so much when I moved to my grandmother” …“I saw blood on the streets, because they used to fight with knives. It was there I learned the knife can be used as a weapon and not just for cooking”.

**Jennifer:**

“I struggled to concentrate at school” … “was a drug and alcohol addict”.

**Deidre:**

“I did drop out of school” … “When I think about my mother, then I just go smoke. I was putting drugs on top of it” … “I saw my dad hit my mom. Then my mother left us, and then when I was older, my stepmom did the same to me and then my boyfriend did do the same to me, they both beat me up” … “Throw my father with a brick against his head and blood was rushing out. I can never forget that”.

**Michelle:**

“That’s why I started to use drugs…I tried to cover it up…the drugs made me forget”.

**Nicole:**

“grumpy” … “swear at everyone” … “rough with him”.

**Jamaal:**

“I was high” … “to live on the streets” … “started smoking glue and breaking into cars….and became a gangster…started robbing people”... “my stepmother did stab me”… “I don’t get the love I needed from my father, so I’m rather going to participate in gangsterism, where I get love and respect”… “beat him to death” … “I took out my knife and I stabbed the one guy to death” …“I was shot”… “beaten up or killed”… “One of the police guys...assaulted my uncle. That is how I became introduced to violence…I saw blood running so he told me “run” and the cops shot me with a rubber bullet”… “never left me” … “jump into my mind”.

**Zubeidah:**

“I was using drugs non-stop from the day before”… “if I was sober, my child would still be alive”.

**Adam:**

“He used to hit my mother in front of us, it was nothing new” … “smacked”… “was murdered” … “I kill a lot of people while in the army…The last one that I shoot was a lady…She was begging but I didn’t have mercy. I kill her…But now, I still have nightmares. This is the price I pay, because she begged not to kill her. At night before I go to sleep, I picture her face and her begging for me not to shoot”…“She was sitting in front of me…I grabbed her…when I strangled her, I didn't think about stopping… I put more force in my arms” … “fight and not stop, just continue, doesn’t matter if it was a woman [he abused his wife] or a young person [he killed his daughter]. When I fight, I’m taught to see nothing in front of me but the person I’m fighting and to hurt you”… “I still have nightmares”

**Ryan:**

“were very violent and fought a lot…and I thought I wanna be like them”…. “went to prison” … “My world shattered…I didn’t care to live anymore…When he passed I started drinking and I got involved with a bad crowd”.

**James:**

“My life changed like really going downhill when my brother passed. We were very close…After that, everything fell apart…My heart died but my brain was working…I started getting involved with the wrong crowd after that” … “My father would run away when I tried to talk to him about how I felt”… “Nothing inside of me feels bad or regret for what I did to that kid…My heart is hard…I don’t get nightmares...I don’t care…You don’t have to do me wrong. If I want to hurt you I do it with the aggression… Kill or be killed” … “I was abused at home so I run away to live on the streets…That is where I get involved in crime…The street life was better than being at home…I’m not evil…I develop this aggression on the streets: for not caring for people, if I want to hurt them, I hurt them…I had to learn to protect myself because the streets is tough and nobody looks out for you besides you” … “I’m used to violence…it’s nothing for me to see someone die…because I have seen it every day. I’m a part of it now”… “steps out of line” … “didn’t listen” to him …“if I ask him to do things for me” … “embarrassing”… “an evil person”… “I started to get angry, because if I see his face, I see his grandmother”…“rubbed it in my face that I don’t have money” … “extremely frustrating” … “you are the first person I have spoken to about this” … “My father told me, when I come inside, and the kids hurt me…he would say: “go out and fight them. Don’t come back here and cry and tell me about kids hurting you””.

**Winnie:**

“She did not have clothes…so I decided instead of her being needy, it is better that I kill her”.

**Howard:**

“While I was a gangster I committed murder ... I take it day by day but it's not easy because when I close my eyes it still replays in my head”… “I’m still so hurt…my father hurt me the most emotionally”.

**Zolu:**

“They beat a guy and took a big stone and put it on top of his head and then he died. I watched them drop that big stone on his head” … “When you come home crying, the men in our family beat you up and they say: “go fight, why you come home crying?””…. “I saw a car hit someone dead…I just look and pass…I don’t have feeling. It’s the way I grow up…I was too young to see and go through those things. So I grow up hard…If you see something you never saw, you will be scared but if you used to see it, you don’t worry about it”.

**Patricia:**

“My heart was very sore, deep down I am emotionally wrecked by my mother”.
